# Enrichment of bacteria samples by centrifugation improves the diagnosis of orthopaedics-related infections via real-time PCR amplification of the bacterial methicillin-resistance gene

**DOI:** 10.1186/s13104-015-1180-2

**Published:** 2015-07-03

**Authors:** Arisa Tsuru, Takao Setoguchi, Naoya Kawabata, Masataka Hirotsu, Takuya Yamamoto, Satoshi Nagano, Masahiro Yokouchi, Hironori Kakoi, Hideki Kawamura, Yasuhiro Ishidou, Akihide Tanimoto, Setsuro Komiya

**Affiliations:** Department of Orthopaedic Surgery, Graduate School of Medical and Dental Sciences, Kagoshima University, Kagoshima, Japan; The Near-Future Locomotor Organ Medicine Creation Course (Kusunoki Kai), Graduate School of Medical and Dental Sciences, Kagoshima University, 8-35-1 Sakuragaoka, Kagoshima, 890-8520 Japan; Infection Control Team, Kagoshima University Hospital, Kagoshima, Japan; Department of Medical Joint Materials, Graduate School of Medical and Dental Sciences, Kagoshima University, Kagoshima, Japan; Molecular and Cellular Pathology, Graduate School of Medical and Dental Sciences, Kagoshima University, Kagoshima, Japan

**Keywords:** Polymerase chain reaction, *mecA* gene, Orthopaedics-related infections, Prosthetic joint infection, Centrifugation

## Abstract

**Background:**

To effectively treat orthopaedic infections by methicillin-resistant strains, an early diagnosis is necessary. Bacterial cultures and real-time polymerase chain reaction (PCR) have been used to define methicillin-resistant staphylococci. However, even when patients display clinical signs of infections, bacterial culture and real-time PCR often cannot confirm infection. The aim of this study was to prospectively compare the utility of real-time PCR for the *mecA* gene detection following centrifugation of human samples with suspected orthopaedic infections.

**Results:**

In addition to the conventional real-time PCR method, we performed real-time PCR following centrifugation of the sample at 4,830×*g* for 10 min in a modified real-time PCR (M-PCR) method. We suspended cultured methicillin-resistant *Staphylococcus aureus* and generated standard dilution series for in vitro experiments. The in vitro detection sensitivity of the M-PCR method was approximately 5.06 times higher than that of the conventional real-time PCR method. We performed bacterial culture, pathological examination, real-time PCR, and M-PCR to examine the infectious fluids and tissues obtained from 36 surgical patients at our hospital. Of these, 20 patients who had undergone primary total hip arthroplasty were enrolled as negative controls. In addition, 15 patients were examined who were clinically confirmed to have an infection, including periprosthetic joint infection (eight patients), pyogenic spondylitis (two patients), infectious pseudoarthrosis (two patients), and after spine surgery (three patients). In one sample from a patient who developed infectious pseudoarthrosis and two samples from surgical site infections after spine surgery, the *mecA* gene was detected only by the M-PCR method. In one patient with infectious pseudoarthrosis, one patient with infection after arthroplasty, and two patients with purulent spondylitis, the detection sensitivity of the M-PCR method was increased compared with PCR (clinical sample average: 411.6 times).

**Conclusions:**

These findings suggest that the M-PCR method is useful to detect methicillin-resistant strains infections. In addition, the centrifugation process only takes 10 min longer than conventional real-time PCR methods. We believe that the M-PCR method could be clinically useful to detect orthopaedic infections caused by methicillin-resistant strains.

**Electronic supplementary material:**

The online version of this article (doi:10.1186/s13104-015-1180-2) contains supplementary material, which is available to authorized users.

## Background

Orthopaedic procedures in particular are associated with a risk of surgical-site infection (SSI) [1]. The incidence of SSI following orthopaedic surgery in Japan is 0.83% for cases of spinal canal stenosis, 0.28% for cases of disc herniation, 0.80% for cases of total hip arthroplasty (THA), and 0.96% for cases of total knee arthroplasty (TKA) [2, 3]. Orthopaedic procedures are also being performed in a growing number of patients with co-morbid conditions such as diabetes mellitus and in increasingly elderly patients, both of which are factors known to increase the risk of SSI [4]. Prosthetic joint infections by multidrug-resistant bacteria comprise one of the most important and complex problems in orthopaedic surgery. The most important and frequently resistant bacteria involved in infection of total joint replacements include methicillin-resistant *Staphylococcus aureus* (MRSA), methicillin-resistant coagulase-negative *Staphylococci*, vancomycin-resistant enterococci, multidrug-resistant *Pseudomonas aeruginosa*, and *Acinetobacter baumannii* [5]. Although culture of samples remains the standard for identifying most organisms causing infection, diagnoses based on culture suffer from a high rate of false negatives caused by insufficient numbers of viable bacteria and effects of previous antibiotic therapy [6]. To resolve these problems, several techniques, including polymerase chain reaction (PCR) detection, have been developed to obtain results faster and more accurately than by using culture methods [7–20]. PCR amplification can detect the *mecA* gene, which gives rise to methicillin-resistance, in orthopaedic prosthetic infections [8, 21–26]. Although real-time PCR methods have been exploited for rapid, sensitive, and reproducible detection [27], sensitivity and specificity of the diagnosis using PCR were 87 and 80% in clinical use, respectively [28]. PCR can theoretically detect *mecA gene* from a single copy of DNA. However, the probability that this single copy would be amplified is low, so many copies are needed to reach a “detection threshold” [13]. We tried to improve the rate of positive identification of the *mecA gene* in these challenging situations. The aim of this cohort study was to compare the utility of real-time PCR for *mecA* gene identification in vitro and in clinical samples following centrifugation of samples. We found that centrifugation of these samples improves detection of the *mecA* gene.

## Results

### M-PCR improved the *mecA* gene detection in vitro

**Figure 1:**
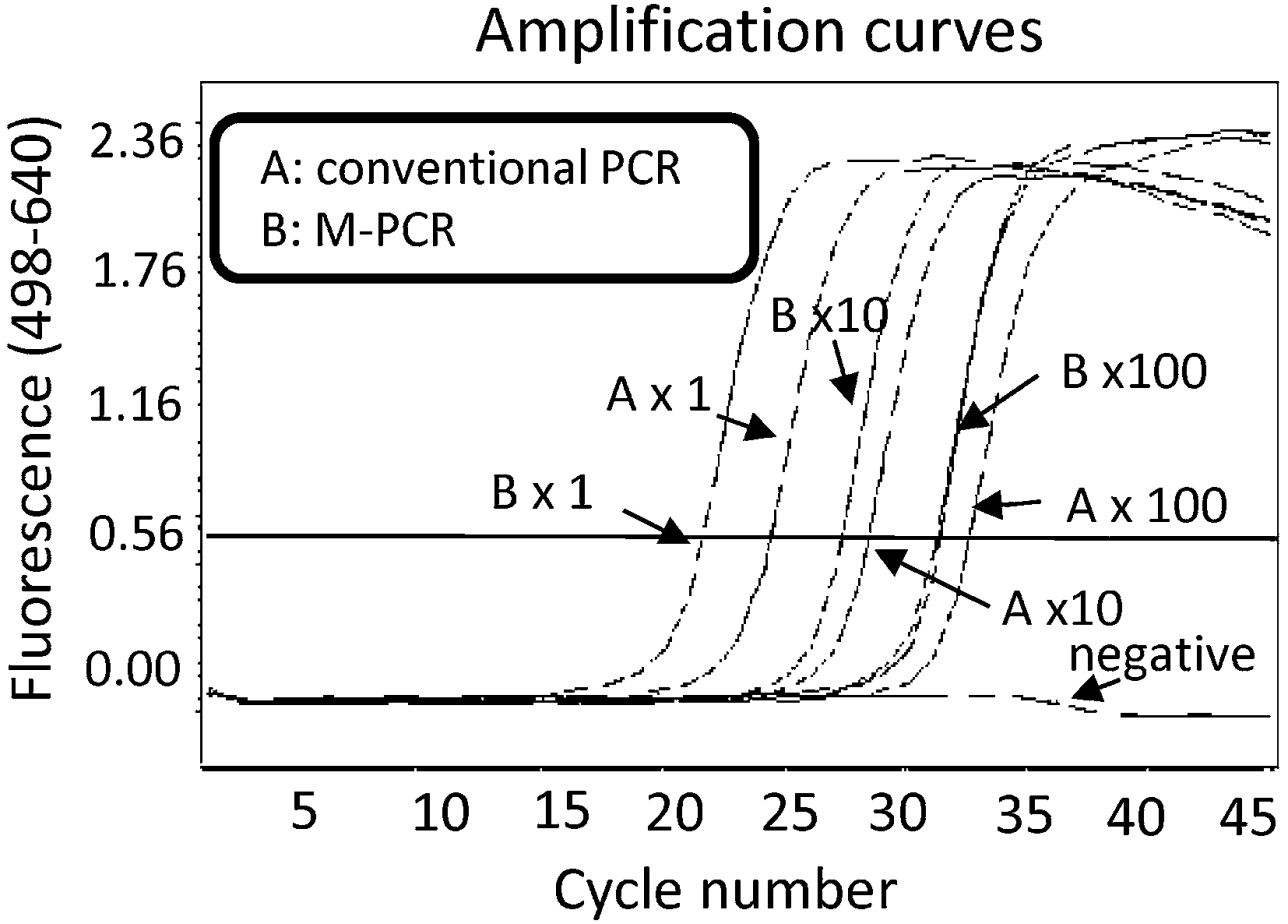
M-PCR improved detection of the *mecA* gene in vitro. To compare the detection sensitivity of the M-PCR method with the conventional real-time PCR method, we created a dilution series using cultured MRSA (×1:1 time; ×10:10 times; ×100:100 times). DNA was purified by conventional methods (*A*) or following centrifugation (*B*: M-PCR). Centrifugation promoted more rapid arrival at the threshold cycle (Fluorescence: 0.56). A comparative C_t_ (ΔC_t_) analysis was performed to examine fold changes of the *mecA* gene. The experiment was performed in triplicate with similar results.

We hypothesized that centrifugation would enrich the number of bacteria in the samples, improving detection of the *mecA* gene of MRS. First, we examined several conditions in which to precipitate cultured MRSA by centrifugation of MRSA-containing phosphate-buffered saline (PBS). We chose to centrifuge at 4,830×*g* for 10 min. Then we performed conventional PCR and M-PCR using the same MRSA dilution series. M-PCR improved detection of the *mecA* gene 5.06-fold over that achieved with conventional real-time PCR methods (Figure 1). There was statistical difference between M-PCR and conventional real-time PCR (p < 0.05).

### M-PCR did not enrich the *mecA* gene of killed bacteria

**Figure 2:**
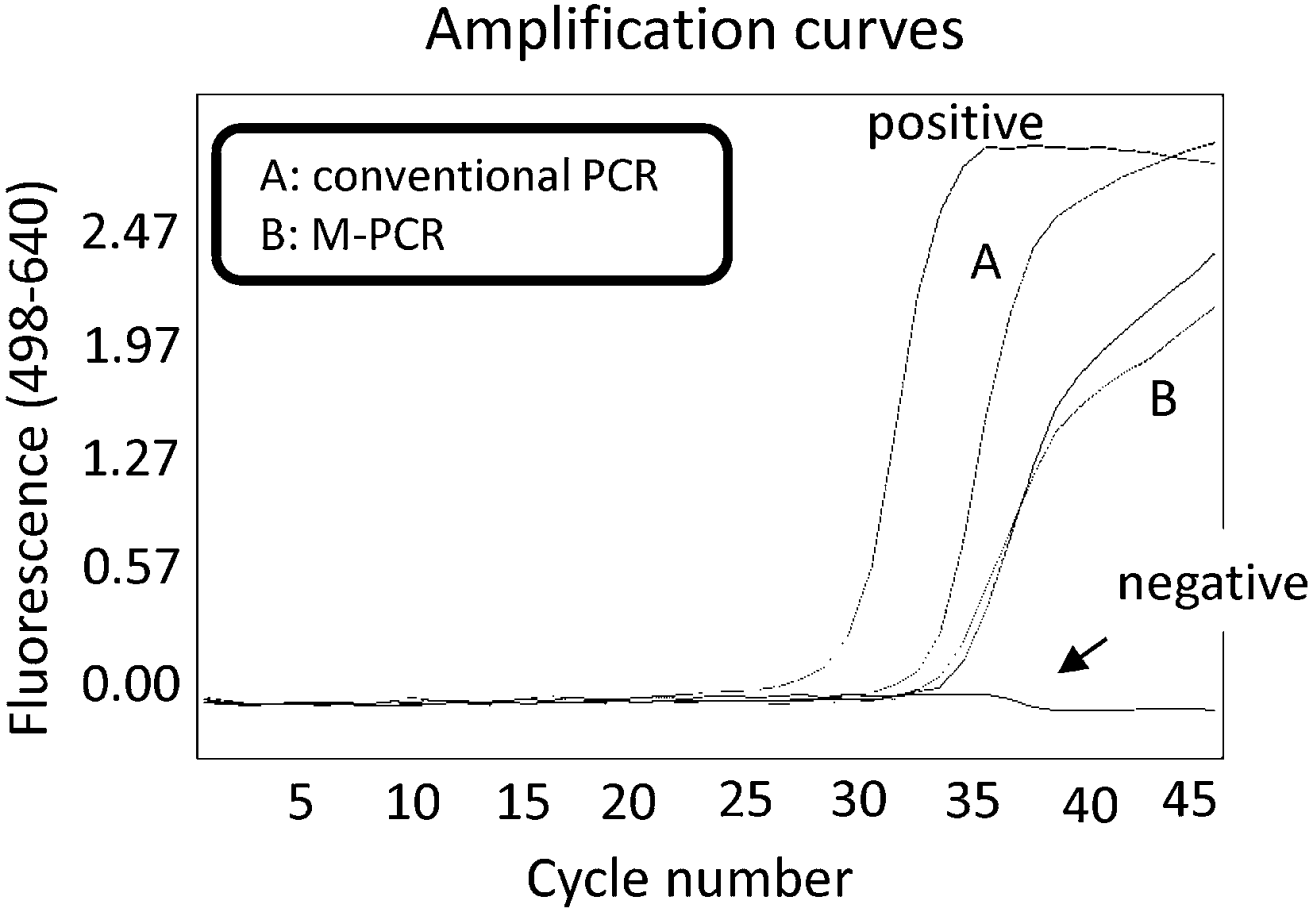
M-PCR did not increase detection of the *mecA* gene in purified DNA. To compare the detection sensitivity of the M-PCR method with the conventional real-time PCR method, we made diluent using the purified DNA of MRSA. DNA was first purified from cultured MRSA and dissolved in 5 mL PBS. Next, DNA was purified by conventional methods (*A*) or following centrifugation (*B*: M-PCR). A comparative Ct (ΔCt) analysis was performed to examine fold changes of the *mecA* gene. The results showed that M-PCR did not increase the detection of the *mecA* gene in purified DNA. The experiment was performed in triplicate with similar results.

It is possible that PCR detects the *mecA* gene from DNA of killed MRS because killed bacteria release genomic DNA into the extracellular environment by autolysis [29]. Theoretically, genomic DNA of MRSA is too small to be precipitated by centrifugation at 4,830×*g* for 10 min. In order to examine whether M-PCR increases the detection of released DNA, DNA was first purified from cultured MRSA by DNA purification. We then performed centrifugation of DNA-containing PBS. M-PCR did not increase the sensitivity for detecting the *mecA* gene (Figure 2). These findings suggest that the released DNA is too small to be precipitated by centrifugation at 4,830×*g* for 10 min.

### M-PCR improved detection of the *mecA* gene in clinical specimens

**Figure 3:**
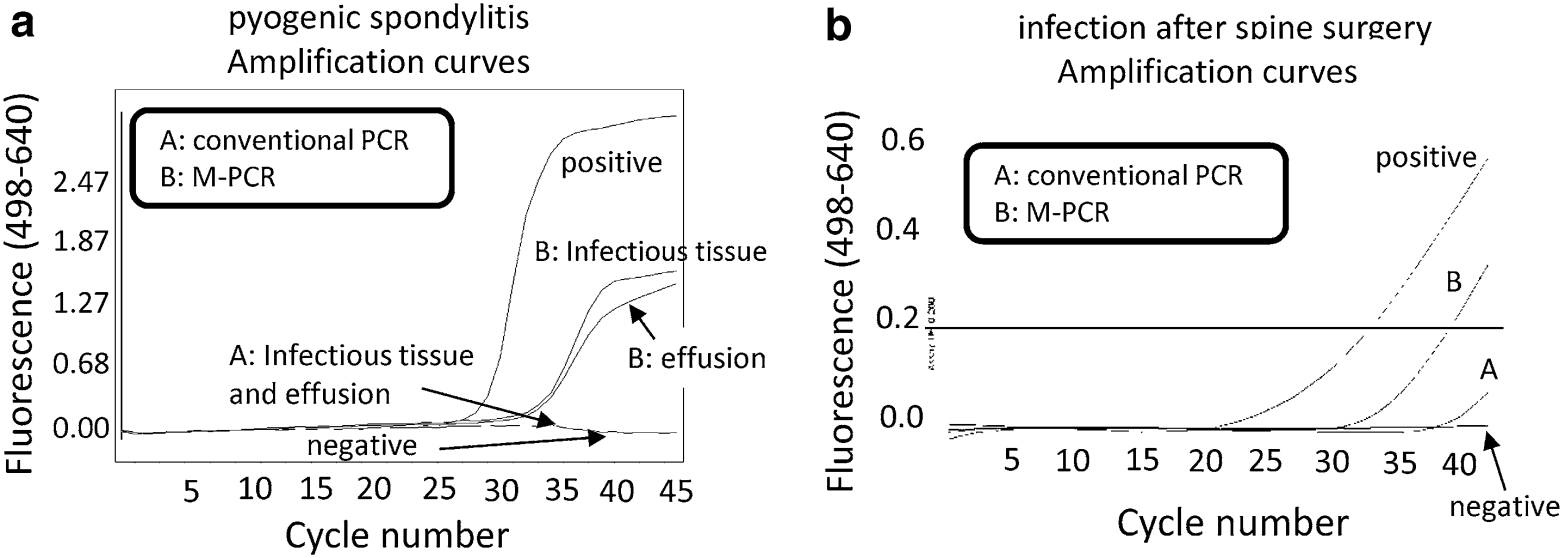
M-PCR improved the detection of *mecA* gene in clinical samples. To compare the detection sensitivity of the M-PCR method with the conventional real-time PCR method, we analysed infectious tissues collected from patients. DNA was purified by conventional methods (*A*) or following centrifugation (*B*: M-PCR). A comparative C_t_ (ΔC_t_) analysis was performed to examine fold changes of the *mecA* gene. Only M-PCR, but not conventional real-time PCR, detected the *mecA* gene from three clinically infected samples, including one pseudoarthrosis (**a**). M-PCR improved the detection of the mecA gene 6.96 times higher than conventional real-time PCR methods (**b**). The experiment was performed in triplicate with similar results.

We analysed the effusion, joint fluid, or infectious tissues from the infectious sites. The demographic data are summarized in Additional file 1: Table S1. Conventional PCR and M-PCR did not detect the *mecA* gene from control primary THA samples. Only M-PCR, and not conventional PCR, detected the *mecA* gene from three clinically infected samples, including one pseudoarthrosis and two surgical site infections following spine surgery (Figure 3). In seven samples, detection of the *mecA* gene was improved by M-PCR. Overall, M-PCR improved detection of the *mecA* gene 411.6 (average) times than was achieved with conventional real-time PCR. There was statistical difference between M-PCR and conventional real-time PCR (p < 0.05). The results for all of the clinical samples are summarized in Additional file 2: Table S2.

## Discussion

The identification of microorganisms is crucial for the treatment of orthopaedic infections. Conventional bacteriological culture of single samples obtained from infectious sites has a high rate of false-positive and false-negative results [30]. Given the limitations of culture, PCR is often used as an additional diagnostic tool for orthopaedic infections [13, 16, 17, 29, 31]. The overall sensitivity in diagnosing prosthetic joint infections increased to 67% when both PCR and bacterial culture were examined together [16]. Mariani et al. reported that although PCR provides greater sensitivity than standard diagnostic tests, such as culture and pathological examinations [25], the sensitivity of PCR was not high enough (50%). To address this, we tried to improve the detection sensitivity of real-time PCR methods. Our findings show that the sensitivity of the M-PCR method was higher than that of the conventional real-time PCR method both in vitro and in clinical samples. No contaminants were detected in our series, and no *mecA* gene DNA was found in samples from patients who had undergone primary total hip arthroplasty (i.e., there were no false-positive results). Several agents are available to treat MRSA, including vancomycin, teicoplanin, and daptomycin. Although detection of the *mecA* gene by PCR could not distinguish between *mecA*-positive coagulase-negative *Staphylococci* and MRSA, these anti-MRS agents can be used to treat infection caused by both strains.

One stage or two-stage re-implantation is considered the standard procedure to treat prosthetic joint infections. Many factors affect the outcomes of patients with implant-related infections, including MRSA infections [32–35]. In addition, Trisha et al. reported that MRSA were isolated in 45% of all prosthetic joint infections [36]. To address this issue, we attempted to improve the detection sensitivity of the *mecA* gene. Our findings show that the M-PCR method improves the detection sensitivity of the *mecA* gene in orthopaedic infections, and it only takes an additional 10 min compared with the conventional real-time PCR method. In addition, it costs very little because most laboratories already own a centrifuge. Enrichment of bacterial DNA by centrifugation for real-time PCR could be useful to evaluate orthopaedic infections, especially after initiating antibiotic therapy, which decreases the number of bacteria.

We showed that M-PCR detected the *mecA* gene from three clinically infected samples that were not detected by conventional PCR. Culture of these samples could not detect the pathogenic bacteria either. We could administer anti-MRSA antibiotics to these three patients and treat the infection based on the M-PCR results. These findings show that M-PCR is clinically useful.

Although real-time PCR may detect DNA released from dead bacteria, our findings suggest that centrifuging purified bacterial DNA did not improve the rate of detecting the *mecA* gene using the M-PCR method. These findings indicate that the released DNA fragments were too short to be precipitated by centrifugation at 4,830×*g* for 10 min.

Several limitations remain to be addressed by the M-PCR approach. Because the number of patients is still low, we do not have enough findings to demonstrate the effectiveness of our M-PCR methods. In addition, centrifugation might increase the collection of bacterial DNA that is still contained within dying or recently dead bacteria. Also, concentrating the specimen may increase the amount of PCR inhibitors and impurities in the extracted DNA and reduce the sensitivity of the PCR. It has been reported that a wide range of bacteria are potentially associated with orthopaedic infections [12, 36]. Further research is required to determine whether M-PCR could be useful to identify other bacteria as well.

## Conclusions

Real-time PCR following centrifugation of samples can improve the detection of the *mecA* gene in orthopaedic infections. It improved the diagnosis of MRS infections and could be used for patients with suspected orthopaedic infections when culture or conventional PCR are negative, and in the diagnosis of patients receiving antimicrobial treatment. Improvements in the *mecA* gene detection by the M-PCR method may help to treat MRS infections in orthopaedic operations, improving patient outcomes and decreasing costs for the hospital.

## Methods

### Bacterium

A cultured methicillin-resistant staphylococcal strain that was obtained from patients with MRSA sinus infections was used for the in vitro portion of this study and was a positive control for *mecA* gene PCR. MRSA was identified by Staphyogram (Terumo, Tokyo, Japan), an identification kit, and the coagulase test. Bacterial colonies were suspended in 5 mL of trypcase soy broth (TSB-ST), 4% NaCl, and 25% glucose. Bacterial culture was performed on a shaker overnight at 37°C.

### Patients

In total, 35 patients hospitalized at the Department of Orthopaedic Surgery of Kagoshima University Hospital in Japan were included in this study. Of these, 20 patients who had undergone primary total hip arthroplasty were used as negative controls. In these controls, infection was excluded by negative results from cultures of three surgical samples and normal C-reactive protein levels, white blood cell counts, and the neutrophil count. In addition, 15 patients were examined who were confirmed to have clinically defined infection, including periprosthetic joint infection (eight patients), pyogenic spondylitis (two patients), infectious pseudoarthrosis (two patients), and after spine surgery (three patients). The diagnosis of orthopaedic infections was made based on clinical presentation, laboratory data, and diagnostic imaging. The clinical symptoms were pain, local swelling and heat, tenderness, aspiration of purulent joint fluid, and purulence surrounding the disease site at the time of surgery [37, 38]. Laboratory findings suggestive of biological inflammatory syndrome were an elevated white blood cell count, erythrocyte sedimentation rate (ESR) (>30 mm/h), and C-reactive protein (CRP) (>1 mg/dL). In addition, synovial fluid or three tissues obtained surgically from the infectious site were cultured and examined histopathologically. These samples were placed in sterile tubes in the operating room and sent to our laboratory for bacterial culture and pathologic examination within 24 h. Culture was performed in the routine work of the hospital clinical microbiology laboratory. The clinical and histopathological aspects of our study were also reviewed by a member of the infectious diseases control team and a pathologist.

### Ethics statement

This research protocol was approved by the Ethics Committee on Clinical Research at Kagoshima University Hospital (Name of research: Enrichment of bacteria samples by centrifugation improves the diagnosis of orthopaedics-related infections via real-time PCR amplification of the bacterial methicillin-resistance gene; No. 25-115).

### DNA extraction from cultured MRSA dilution series

We prepared two dilution series using culture solutions of MRSA. A total of 10 mg of a MRSA colony-containing agar was dissolved in 5 μL of culture medium and then cultured overnight. Then 500 μL of culture medium was added to 4,500 μL of PBS. The tenfold dilution series was prepared using PBS. For conventional PCR, DNA was purified from this dilution series using a QIAamp DNA Mini kit (QIAGEN) as per the manufacturer’s recommendations. Dilution series samples were centrifuged at 4,830×*g* for 10 min before DNA extraction for the M-PCR technique. The supernatants were discarded and the pellets dissolved in 200 µL of PBS. The DNA was purified using a QIAamp DNA Mini kit as per the manufacturer’s recommendations.

### DNA extraction from killed MRSA

For the killed bacterium DNA detection assay, DNA was first purified from cultured MRSA using a DNA purification kit (QIAamp DNA Mini kit) as per the manufacturer’s recommendations. We then dissolved a purified DNA by PBS. Samples were centrifuged at 4,830×*g* for 10 min before DNA extraction for the M-PCR technique. The supernatants were discarded, and the pellets were dissolved in 200 µL PBS, as before. Finally, DNA was purified using a QIAamp DNA Mini kit.

### DNA extraction from clinical specimens

Clinical samples (0.4 g) were divided into smaller pieces by scissors. These cleaved samples were dissolved in 10 mL of PBS. Sonication was then performed at 20 kHz for 10 min (Tomy Seiko Co. Ltd., Tokyo, Japan). For conventional PCR, 200 μL of the sample containing PBS was used. For M-PCR, 9,800 μL of the sample containing PBS was centrifuged at 4,830×*g* for 10 min before DNA extraction. The supernatants were discarded and the pellets dissolved in 200 µL PBS. DNA was extracted following the manufacture’s recommendations (QIAamp DNA Mini kit). DNA extracts were reconstituted in a final volume of 50 µL and stored at −20°C.

### Real-time PCR

The extracted DNA samples were examined by real-time PCR using the LightCycler^®^ system (Roche Diagnostics, Mannheim, Germany). The LightCycler MRSA Detection Kit provides primers and probes for amplification and sequence-specific detection of the *mecA* gene. Five microlitres of the extracted DNA elution was added to the master mix at a final volume of 20 µL for each reaction. The real-time PCR reaction was performed following the kit manufacture’s recommendations. Briefly, the cycling conditions were a hot start at 95°C for 10 min, followed by 45 cycles of denaturation at 95°C for 10 s, annealing at 55°C for 10 s, and extension at 72°C for 12 s.

We also synthesized our own primers for the *mecA* gene and performed real-time PCR because the LightCycler^®^ MRSA Detection Kit has been discontinued. We used the Eco™ Real-time PCR System (Illumina, San Diego, CA, USA), and the primer was designed for the *mecA* gene as previously reported [39]. The forward and reverse primer sequences were: 5′-TGCTATCCACCCTCAAACAGG-3′ and 5′-AACGTTGTAACCACCCCAAGA-3′. The PCR mixtures totalled 20 μL per reaction, and consisted of 10 μM concentration of each primer and 10 μL of 2× Fast SYBR^®^ Green Master Mix (Life Technologies Corporation, Carlsbad, SD). We added 2 μL of the extracted DNA to the PCR mixture. Reaction mixtures were incubated at 95°C for 20 s followed by 40 cycles of 95°C for 3 s and 60°C for 20 s. We used the cultured MRSA as the positive control and nuclease-free water as the negative control.

### Statistical analysis

A Statistical differences between groups were assessed by Mann–Whitney’s U test (Microsoft, Albuquerque, NM, USA). All statistical analyses were performed using and Excel Statics 2012 (SSRI, Osaka, Japan).

### Patient consent

Written informed consent was obtained from patients and their families to publish this report. Copies of the written consent forms are available for review by the Editor-in-Chief of the journal.

## Additional files

Additional file 1:
**Table S1.** Clinicopathological data of the patients. Grey lines show the positive findings for infection. Additional file 2:
**Table S2.** Summary of the clinical results.

## Abbreviations

PCR: polymerase chain reaction
M-PCR: modified polymerase chain reaction
MRSA: methicillin-resistant *Staphylococcus aureus*
PBS: phosphate-buffered saline
ESR: erythrocyte sedimentation rate
CRP: C-reactive protein
