# Supplementary material for: Enrichment of bacteria samples by centrifugation improves the diagnosis of orthopaedics-related infections via real-time PCR amplification of the bacterial methicillin-resistance gene
Source: BMC Res Notes. 2015 Jul 3;8:288. doi: 10.1186/s13104-015-1180-2 (PMC4490765; doi:10.1186/s13104-015-1180-2)
Supplement: Additional file 1: — Table S1. Clinicopathological data of the patients. Grey lines show the positive findings for infection. [file 13104_2015_1180_MOESM1_ESM.docx]

|  | gender | age | primary disease | culture results | PMN 5≥ | PCR | M-PCR | W.B.C.  (cell count/ml) | CRP(mg/dl) | pyrexia/ local swelling, heat, or tenderness | pus |
| --- | --- | --- | --- | --- | --- | --- | --- | --- | --- | --- | --- |
| 1 | male | 69 | pyogenic spondylitis | MRSA | ＋ | ＋ | ＋ | 8010 | 20.7 | + | + |
| 2 | female | 66 | pyogenic spondylitis | － | － | ＋ | ＋ | 4500 | 5.57 | - | - |
| 3 | male | 49 | infectious pseudoarthrosis | － | － | － | ＋ | 4830 | 2.37 | + | + |
| 4 | male | 67 | infectious pseudoarthrosis | － | ＋ | ＋ | ＋ | 10200 | 4.46 | + | + |
| 5 | female | 54 | lumber disc herniation | S.spicies | ＋ | － | ＋ | 5010 | 4.08 | + | + |
| 6 | male | 18 | spine tumor | S.spicies | ＋ | － | ＋ | 6970 | 4.02 | - | + |
| 7 | male | 20 | scoliosis | S.aureus | － | － | － | 4900 | 2.45 | - | + |
| 8 | female | 73 | Periprosthetic joint infection | － | － | － | － | 13960 | 17.1 | + | + |
| 9 | female | 86 | Periprosthetic joint infection | － | － | ＋ | ＋ | 10870 | 21.8 | + | + |
| 10 | male | 57 | Periprosthetic joint infection | E.faecalis | ＋ | ＋ | ＋ | 7040 | 2.55 | + | + |
| 11 | female | 88 | Periprosthetic joint infection | － | ＋ | － | － | 4150 | 0.29 | - | - |
| 12 | male | 69 | Periprosthetic joint infection | － | ＋ | － | － | 4200 | 5.22 | - | + |
| 13 | female | 85 | Periprosthetic joint infection | － | － | － | － | 4880 | 0.82 | + | - |
| 14 | female | 83 | Periprosthetic joint infection | E.coli | ＋ | － | － | 5630 | 0.29 | + | + |
| 15 | female | 61 | Periprosthetic joint infection | － | ＋ | － | － | 3860 | 0.15 | - | - |

Table S1
