# Supplementary material for: Enrichment of bacteria samples by centrifugation improves the diagnosis of orthopaedics-related infections via real-time PCR amplification of the bacterial methicillin-resistance gene
Source: BMC Res Notes. 2015 Jul 3;8:288. doi: 10.1186/s13104-015-1180-2 (PMC4490765; doi:10.1186/s13104-015-1180-2)
Supplement: Additional file 2: — Table S2. Summary of the clinical results. [file 13104_2015_1180_MOESM2_ESM.docx]

Table S2

|  | Detection of MRSA  by Microbiologic culture | Detection of neutrophil infiltration  by histopathologic evaluation | Detection of  The *mecA* gene  by conventional PCR | Detection of the *mecA* gene  by M-PCR |
| --- | --- | --- | --- | --- |
| Non-infectious tissue  20 patients | 0/20 | 0/20 | 0/20 | 0/20 |
| Infectious tissue  15 patients | 1/15 | 9/15 | 5/15 | 8/15 |
